# Realistic high-resolution lateral cephalometric radiography generated by progressive growing generative adversarial network and quality evaluations

**DOI:** 10.1038/s41598-021-91965-y

**Published:** 2021-06-15

**Authors:** Mingyu Kim, Sungchul Kim, Minjee Kim, Hyun-Jin Bae, Jae-Woo Park, Namkug Kim

**Affiliations:** 1 Department of Convergence Medicine, Asan Medical Center, College of Medicine, University of Ulsan, 88 Olympic-ro 43-gil, Songpa-gu, Seoul, 05505 Republic of Korea; 2 Department of Biomedical Engineering, Asan Medical Institute of Convergence Science and Technology, Asan Medical Center, College of Medicine, University of Ulsan, Seoul, Republic of Korea; 3 Department of Orthodontics, Kooalldam Dental Hospital, 1418 Kyoungwondaero, Bupyong-gu, Incheon, 21404 Republic of Korea; 4 Department of Radiology, Asan Medical Center, College of Medicine, University of Ulsan, Seoul, Republic of Korea

**Keywords:** Experimental models of disease, Information technology, Computer science

## Abstract

Realistic image generation is valuable in dental medicine, but still challenging for generative adversarial networks (GANs), which require large amounts of data to overcome the training instability. Thus, we generated lateral cephalogram X-ray images using a deep-learning-based progressive growing GAN (PGGAN). The quality of generated images was evaluated by three methods. First, signal-to-noise ratios of real/synthesized images, evaluated at the posterior arch region of the first cervical vertebra, showed no statistically significant difference (t-test, p = 0.211). Second, the results of an image Turing test, conducted by non-orthodontists and orthodontists for 100 randomly chosen images, indicated that they had difficulty in distinguishing whether the image was real or synthesized. Third, cephalometric tracing with 42 landmark points detection, performed on real and synthesized images by two expert orthodontists, showed consistency with mean difference of 2.08 ± 1.02 mm. Furthermore, convolutional neural network-based classification tasks were used to classify skeletal patterns using a real dataset with class imbalance and a dataset balanced with synthesized images. The classification accuracy for the latter case was increased by 1.5%/3.3% at internal/external test sets, respectively. Thus, the cephalometric images generated by PGGAN are sufficiently realistic and have potential to application in various fields of dental medicine.

## Introduction

At present, many clinicians use cephalometric analyses to better understand the underlying basis of a malocclusion^1,2^. A cephalometric analysis consists of identifying landmarks that represent important facial-structure points and evaluating the distances or angles between the landmarks and the lines that compose the landmarks^3–5^. This enables a quantification of the relationship between the facial bone structure and the teeth. However, because of the over-simplified and omitted measurement details in malocclusion research, clinical applications for diagnosing all types of malocclusion are inadequate^6^. Hence, in addition to comparing individual measurements with a normal image, many clinicians also evaluate anatomical patterns, including soft tissue^7^.

Recently, owing to progress in the computer-vision field and the rapid development of deep learning, many studies have investigated the automated diagnosis of cephalometric X-ray images using deep learning. Yu et al.^8^ classified patients' structural patterns using deep learning, without using cephalometric tracing information. They achieved a mean accuracy of over 90% for skeletal pattern (i.e., Class I, Class II, Class III) classification. However, they trained the model with a dataset that excluded ambiguous skeletal classes. These unclear patient-selection criteria could make the model inapplicable in actual clinical settings. Lee et al.^9^ proposed a method to classify patients for successful treatment by orthodontics or maxillofacial surgery. However, because information regarding the patients’ skeletal features was absent, their method is difficult to apply in an actual clinical setting.

The aforementioned issues are a result of insufficient data to enable deep-learning models to learn sufficient anatomical structures to discriminate pattern differences, and also the significantly low amount of data for the abnormal skeletal patterns used to learn various occlusion patterns^10^. Augmenting images using geometric transforms or intensity variations are advances that can be applied to solve the mentioned issues^11,12^. However, geometric and intensity augmentations (e.g., translation, rotation, scaling, and filtering) do not improve performance because these types of transforms do not significantly change their intrinsic image properties.

To address this issue, generative adversarial networks (GANs)^13^ have been widely used to synthesize infinitely unique images using unsupervised methods^2,14^. Few studies have applied GANs to generate realistic images in the clinical region. Frid-Adar et al.^2^ synthesized computed tomography (CT) images around liver lesions using a GAN. They performed a convolutional neural network (CNN) classification between a dataset that was classically augmented using geometric or intensity transformations and a dataset that was synthetically augmented using a GAN. The classification performance using synthetic augmentation was 5% better in terms of sensitivity and specificity. This study shows that using synthetic images for data augmentation can overcome the small-dataset problem.

Sandfort, et al.^14^ used CycleGAN^15^ to generate non-contrast CT images from contrast CT images. They augmented the dataset by combining contrast CT and synthesized non-contrast CT images. Then, they assessed the segmentation performance on organs, e.g., kidney, liver, and spleen. The average performance showed a Dice score of 0.747, which is considerably higher than that of using a dataset containing only contrast CT images (Dice score of 0.101). Consequently, synthesizing images using GANs has potential for various applications, such as data augmentation of various disease cases to increase CNN performance, diagnostic assistance, treatment planning, and physician training.

In this study, lateral cephalometric images were trained to synthesize realistic images. Among the various types of GANs available, a progressive-growing GAN (PGGAN)^16^ was chosen. Validations were performed to evaluate quality and utility. For the quality evaluation, signal-to-noise ratio (SNR) calculation on the posterior region of the first cervical vertebra, image Turing test, and landmark tracing were performed. In terms of utility, CNN-based classification task was performed to validate whether the class balanced dataset by adding synthesized images could be used for increasing performance in a real cephalometric dataset with an intrinsic class imbalance issue.

## Methods

### Training-data collection

A total of 19,152 cephalometric images of patients who received orthodontic treatment between 2009 and 2019 were obtained (institutional review board (IRB) No: P01-202011-21-032) from the Kooalldam Dental Hospital in Korea. From this total, 3319 poor quality images that were used for testing equipment were excluded. Finally, 15 833 images were used for the PGGAN training. The mean age of the patients was 25.7 ± 7.2 years ranging from 19 to 76 years and 35% were male.

This retrospective study was conducted according to the principles of the Declaration of Helsinki and was performed in accordance with current scientific guidelines. The study protocol was approved by the IRB of the Korea National Institute for Bioethics Policy, Seoul, Korea. Informed consent was acquired from all the patients and from 13 readers who participated in the image Turing test.

### PGGAN training

**Figure 1:**
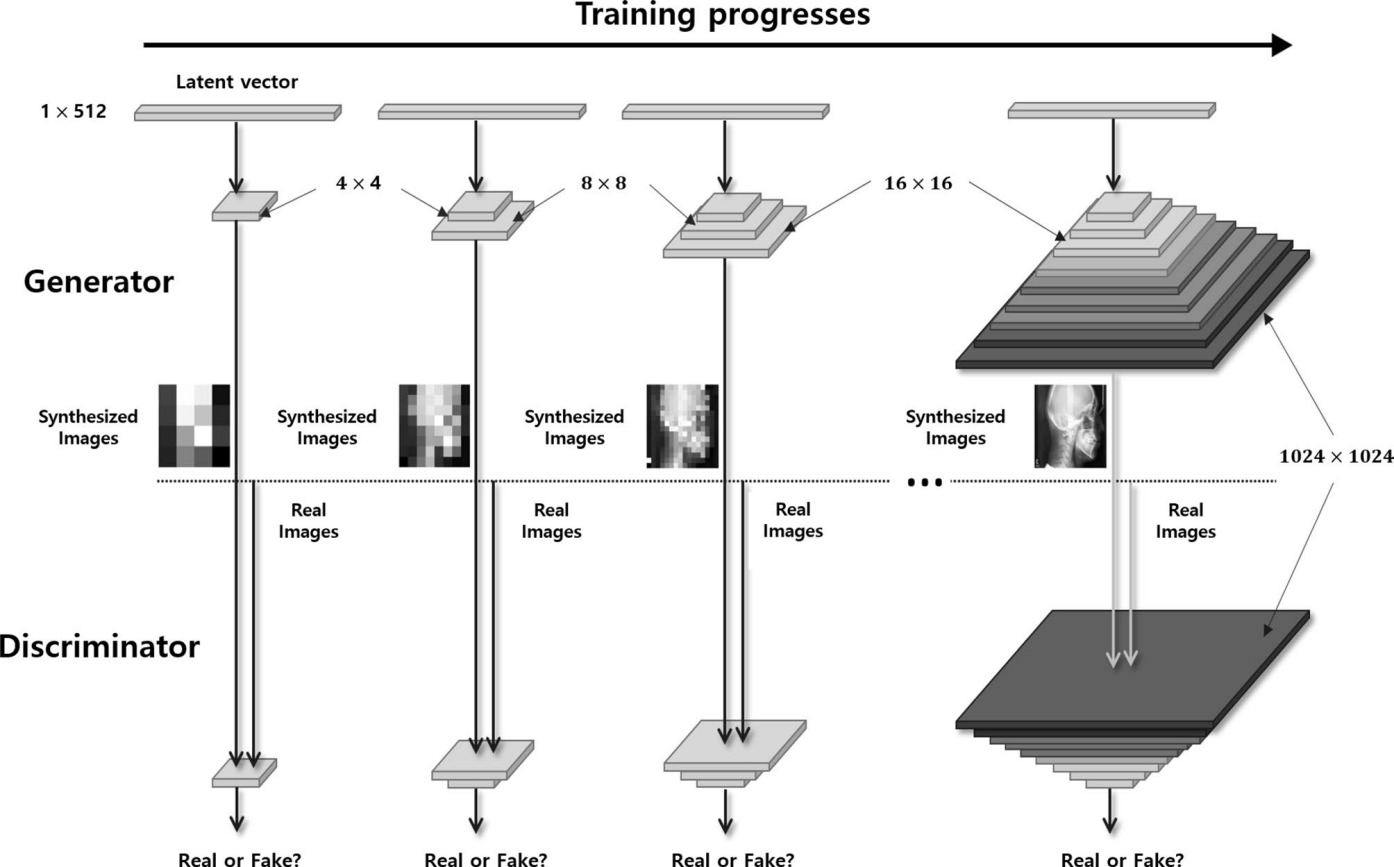
Conceptual architecture of PGGAN for training. PGGAN synthesizes images from a low resolution (4 × 4 pixels) to a high resolution (1024 × 1024 pixels) by adding layers to the network.

PGGAN is a variant of GAN architecture with a different training method. The traditional GAN has two networks, a generator and a discriminator. These two networks act in an adversarial manner: the generator produces a synthesized image and the discriminator indicates whether this image is real or not. The distinctive characteristic of PGGAN training is that the generator generates images progressively. Both GAN and PPGAN progressively grow starting from a low resolution (4 × 4 pixels) to a high resolution (1024 × 1024 pixels) by adding layers to the network, as shown in Fig. 1. This method enables a stable training by learning from easier images with coarse structure to difficult ones with fine details. PGGAN was chosen for cephalometric image generation because this model performed better in reconstructing global structures and fine details with a high-resolution quality among other GAN variant models^17–19^.

**Figure 2:**
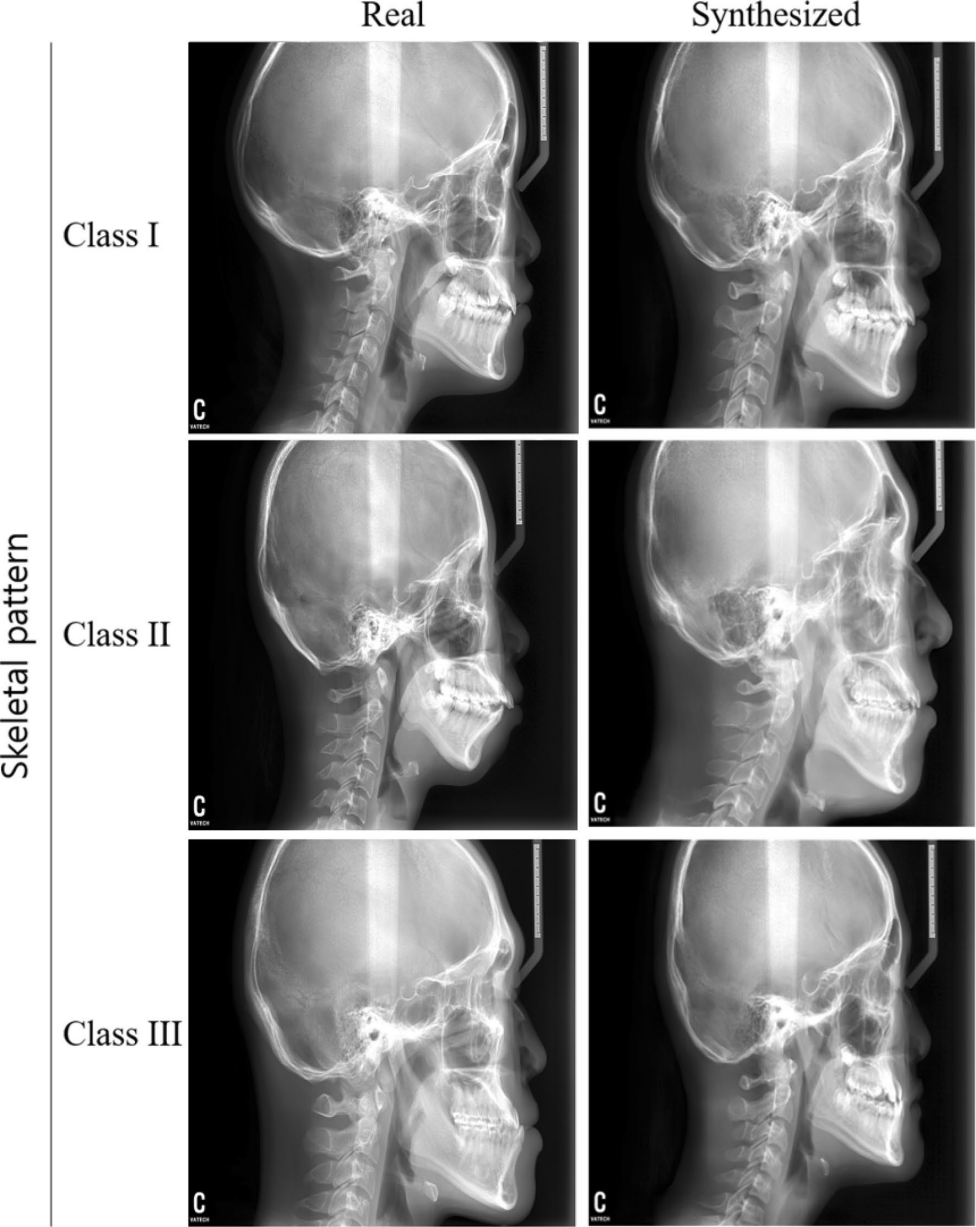
Examples of real and synthesized images for each skeletal pattern.

The 15 833 lateral cephalograms were used to train the PGGAN in an unsupervised manner. The input images were resized from 1880 × 2360 to 1024 × 1024 pixels without considering the aspect ratio. Two Titan-RTX 24-GB graphics processing units (GPUs) were used, the learning rate was set to 0.001, and other parameters were fixed as default. Consequently, synthesized images were produced, and metric evaluation, image Turing test, landmark tracing, and augmentation efficacy test were performed to validate the model. Here, synthetic cephalometric X-ray images generated by GAN would have a distribution similar to that of a real dataset including gender, age distribution, imaging parameters, and X-ray machines, as GAN is known to train the distribution of the training dataset. As an example, comparisons between real and synthesized images with skeletal pattern are shown in Fig. 2. Here, the PGGAN was used from a public website (Tensorflow-gpu 1.6.0, Python 3.4.0).

### Signal-to-noise ratio measurements

SNRs were measured for 100 images (50 real and 50 synthesized) to evaluate whether the contrast of anatomically distinct features in cephalometric radiography show consistency between real and synthesized images. Here, 50 real images were randomly selected from the PGGAN training set and 50 synthesized images were randomly generated by the trained PGGAN. The distinct feature was chosen at the posterior arch of the first cervical vertebra because it is clearly defined to all images. For the noise estimation, tissue regions at the posterior direction of the posterior arch of the first cervical vertebra were chosen to avoid interference with other bone regions. The signal and noise regions of the 100 images were manually segmented by M. Kim. An example of segmented image is shown in Supplementary Appendix Fig. S2. For each image, the signal was calculated by taking the mean of the posterior arch of the first cervical vertebra region. The noise was calculated by taking one standard deviation (SD) of 10 × 10 pixels with center at a manually segmented region. Finally, the SNR was estimated by taking the ratio of the signal and the noise. We conducted a t-test of SNRs between the real and synthesized images to evaluate the statistical differences. Here, the R statistical environment, version 3.5.3, was used for the statistical analysis, with a significance level of p < 0.05.

### Image Turing test

For the image Turing test, we prepared the 100 images used for SNR measurement. The image Turing test was conducted with 13 readers by displaying images one-by-one through a dedicated web-based interface. Two of the readers were dental students, four were dental residents, and seven were dental specialists. The dental residents consisted of two non-orthodontic and two orthodontic residents. The dental specialists consisted of two non-orthodontic specialists, two orthodontists with 10 years of clinical experience, and three with 20 years of clinical experience. We divided the readers into two groups, non-orthodontists (Group 1) and orthodontists (Group 2), and compared their results.

To reduce the environmental variability during the image Turing test, the images were displayed in the same order and earlier answers were prohibited. Readers were informed that there were 50 real and 50 synthesized test images. In addition, none of the readers had experienced synthesized images before the test. All readers successfully finished the test. The sensitivity, specificity, and accuracy were derived for evaluation after the test. Here, we define a real image as positive and a synthesized image as negative. The inter-reader agreement of the image Turing test was evaluated using the Fleiss Kappa^20^.

### Cephalometric tracing on synthesized images

Cephalometric tracing by identifying landmarks is important step for orthodontic diagnosis and treatment planning. To use synthesized image on augmentation purposes for improving deep learning models and other clinical situations, landmarks containing clinical information should be identified accurately. To verify the position recognition rate of landmarks, a total of 42 landmarks were traced by two orthodontists (J. Park and S. E. Jang) on the 50 synthesized images used for SNR measurement. The orthodontists knew that the cephalometric images were synthesized. They traced the landmarks according to their anatomical definitions. A cephalometric image with the landmark positions is shown in Supplementary Appendix Fig. S1 and their names are shown in Supplementary Appendix Table S1. We compared each point of traced landmark differences between the two readers. Then, the average difference was calculated and different landmark points were discussed.

### Efficacy of generated images as augmentation for class imbalanced dataset

To verify the utility of the synthesized images, a CNN-based classification task was performed. The task consisted of classifying skeletal patterns (i.e., Class I, Class II, and Class III) with and without adding synthesized images for balancing the intrinsically imbalanced dataset. Our hypothesis was that if synthesized images contained clinically important information, the augmentation could increase classification performance.

The dataset was obtained from the Department of Orthodontics in 10 multi-centers in Korea. The distribution of skeletal patterns is 601 for Class I, 490 for Class II, and 553 for Class III, which has not a significant class imbalance. The skeletal patterns were classified on the A point-Nasion-B point angle. The dataset was divided for training and internal test with ratio of 9:1. In addition, 181 skeleton patterns from eight medical centers in Korea were prepared as an external test set.

Synthesized images were also prepared for balancing the number of images in the real dataset. Thus, 3000 synthesized images were randomly generated using trained PGGAN. Then, their skeletal patterns were classified using the model developed in this study using only real data set. The number of synthesized images classified in each class were 1550, 765, and 685, for Classes I, II, and III, respectively. To overcome classification error and increase classification accuracy, synthesized images among the 3000 were chosen with likelihood criteria of model output. With the likelihood of 0.9 criteria, 940, 435, and 483 images were collected for Classes I, II, and III, respectively. Among them, 299, 410, and 347 images were added to Classes I, II, and III, respectively, to balance the class imbalance in the real dataset. Finally, 740 images in each class were set up for model training.

For the training, vanilla DenseNet-169^21^ was used. Training was performed using two datasets: the real dataset with intrinsic class imbalance and a class balanced dataset with addition of synthesized images. For the model validation, both internal test and external tests were performed and their sensitivity, specificity, and accuracy were derived. Here, pytorch version 3.6 and tensorflow version 2.3 were used for the development.

The study protocol was reviewed and approved by the IRB of SNUDH (ERI20022), Korea National Institute for Bioethics Policy for KADH (P01-202010-21-020), Ajou University Hospital Human Research Protection Center (AJIRB-MED-MDB-19-039), AMC (2019-0927), CNUDH (CNUDH-2019-004), CSUDH (CUDHIRB 1901 005), EUMC (EUMC 2019-04-017-003), KHUDH (D19-007-003), KNUDH (KNUDH-2019-03-02-00), and WKUDH (WKDIRB201903-01).

## Results

The SNRs for the real and synthesized images were 23.54 ± 10.80 and 26.46 ± 12.13, respectively, and the t-test showed no statistically significant difference (p = 0.211) between the SNR values.

**Figure 3:**
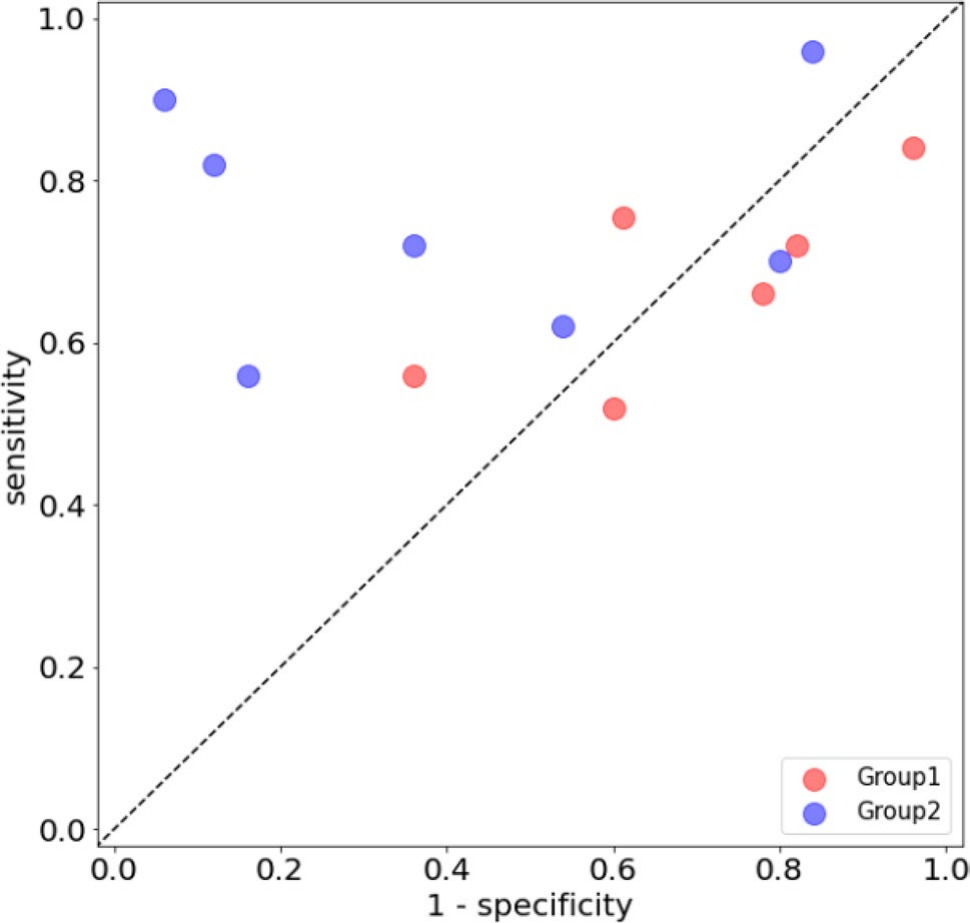
Human performance in the image Turing test.

The results of the image Turing test are shown in Table 1, with mean accuracy, sensitivity, and specificity of the readers. The results of each reader are presented in Supplementary Appendix Table S2. The sensitivities of Groups 1 and 2, which were 67.6 ± 11.0 and 75.4 ± 13.5, respectively, were not significantly different. In contrast, the specificity of Group 1 (31.1 ± 19.2) was considerably lower than that of Group 2 (58.9 ± 29.9). As a result, the mean accuracy of Group 1 was lower than that of Group 2. The sensitivity *versus* specificity for each reader can be visualized in Fig. 3. The mean Fleiss Kappa was 0.023 for Group 1 and 0.109 for Group 2, which indicates that the classification inter-rater agreement was poor for both groups.

**Table 1:** Average assessment results for readers in each group.

	Group 1	Group 2	Overall
**Total**			
Accuracy (%)	49.2 ± 6.4	67.1 ± 15.7	58.8 ± 15.2
Sensitivity (%)	67.6 ± 11.0	75.4 ± 13.5	71.8 ± 13.0
Specificity (%)	31.1 ± 19.2	58.9 ± 29.9	46.1 ± 29.0

The landmark-position differences between the two orthodontists are shown in Table 2. The average difference was 2.08 ± 1.02 mm. The landmark position with the largest difference was the occlusal plane point (5.95 ± 2.42 mm).

**Table 2:** Inter-reader identified-landmark position difference for 42 landmarks.

Landmark name	Intra-observer difference in mm (Mean ± SD)		
	Δx	Δy	$\sqrt{{\mathrm{\Delta x}}^{2}+{\mathrm{\Delta y}}^{2}}$
A-Point	0.78 ± 0.69	2.12 ± 1.32	2.38 ± 1.29
Anterior nasal spine	1.70 ± 1.23	0.76 ± 1.13	2.07 ± 1.40
Articulare	0.46 ± 0.55	0.68 ± 0.57	0.92 ± 0.67
B-point	0.60 ± 0.43	1.18 ± 1.10	1.44 ± 1.03
Basion	1.16 ± 0.98	1.78 ± 1.99	2.29 ± 2.05
Columella	0.89 ± 0.70	0.44 ± 0.38	1.03 ± 0.75
Corpus left	3.30 ± 2.22	1.40 ± 1.71	3.69 ± 2.67
Glabella	0.35 ± 0.30	2.49 ± 2.12	2.57 ± 2.07
Hinge axis	0.69 ± 0.71	1.39 ± 1.12	1.62 ± 1.24
Labrale superius	0.61 ± 0.51	0.82 ± 0.65	1.09 ± 0.73
Lower lip	0.44 ± 0.32	0.82 ± 0.64	1.02 ± 0.59
Mandible 1 crown	0.38 ± 0.33	0.37 ± 0.35	0.58 ± 0.42
Mandible 1 root	1.62 ± 1.00	1.97 ± 1.20	2.63 ± 1.42
Mandible 6 distal	1.04 ± 1.67	0.47 ± 0.60	1.24 ± 1.70
Mandible 6 root	1.78 ± 1.70	0.86 ± 0.84	2.11 ± 1.75
Maxilla 1 crown	0.34 ± 0.27	0.25 ± 0.19	0.47 ± 0.26
Maxilla 1 root	0.88 ± 0.63	1.35 ± 0.84	1.70 ± 0.90
Maxilla 6 distal	0.96 ± 1.43	0.79 ± 0.61	1.37 ± 1.44
Maxilla 6 root	1.59 ± 1.57	0.99 ± 0.67	2.03 ± 1.52
Menton	0.71 ± 0.48	0.15 ± 0.12	0.75 ± 0.46
Nasion	0.44 ± 0.64	0.79 ± 0.88	1.00 ± 1.00
Occlusal plane point	5.87 ± 2.43	0.76 ± 0.54	5.95 ± 2.42
Orbitale	1.02 ± 0.76	0.78 ± 0.62	1.39 ± 0.83
PM	0.58 ± 0.34	1.09 ± 0.83	1.35 ± 0.71
Pogonion	0.47 ± 0.33	1.35 ± 1.33	1.53 ± 1.26
Porion	0.99 ± 0.64	0.63 ± 0.46	1.29 ± 0.58
Posterior nasal spine	1.33 ± 0.77	0.39 ± 0.34	1.47 ± 0.69
Pronasale	0.29 ± 0.27	0.98 ± 0.77	1.08 ± 0.73
Pterygoid	0.79 ± 0.65	1.25 ± 1.07	1.60 ± 1.08
R1	1.50 ± 1.22	4.09 ± 2.51	4.62 ± 2.34
R3	1.34 ± 1.18	4.11 ± 1.81	4.44 ± 1.90
Ramus down	0.93 ± 0.67	3.70 ± 2.72	3.95 ± 2.62
Sella	0.44 ± 0.35	0.28 ± 0.22	0.59 ± 0.31
Soft tissue A	0.57 ± 0.51	1.51 ± 0.86	1.67 ± 0.89
Soft tissue B	0.60 ± 0.54	1.67 ± 1.27	1.85 ± 1.27
Soft tissue menton	1.19 ± 1.09	0.48 ± 0.54	1.36 ± 1.13
Soft tissue nasion	0.60 ± 0.53	1.30 ± 1.16	1.52 ± 1.17
Soft tissue pogonion	1.62 ± 1.99	5.46 ± 4.66	5.76 ± 5.00
Stmi	0.92 ± 0.70	0.44 ± 0.36	1.11 ± 0.65
Stms	1.76 ± 0.87	0.44 ± 0.33	1.84 ± 0.87
Subnasale	0.76 ± 0.65	0.44 ± 0.31	0.94 ± 0.65
Upper lip	0.44 ± 0.47	1.37 ± 0.97	1.50 ± 0.99

**Figure 4:**
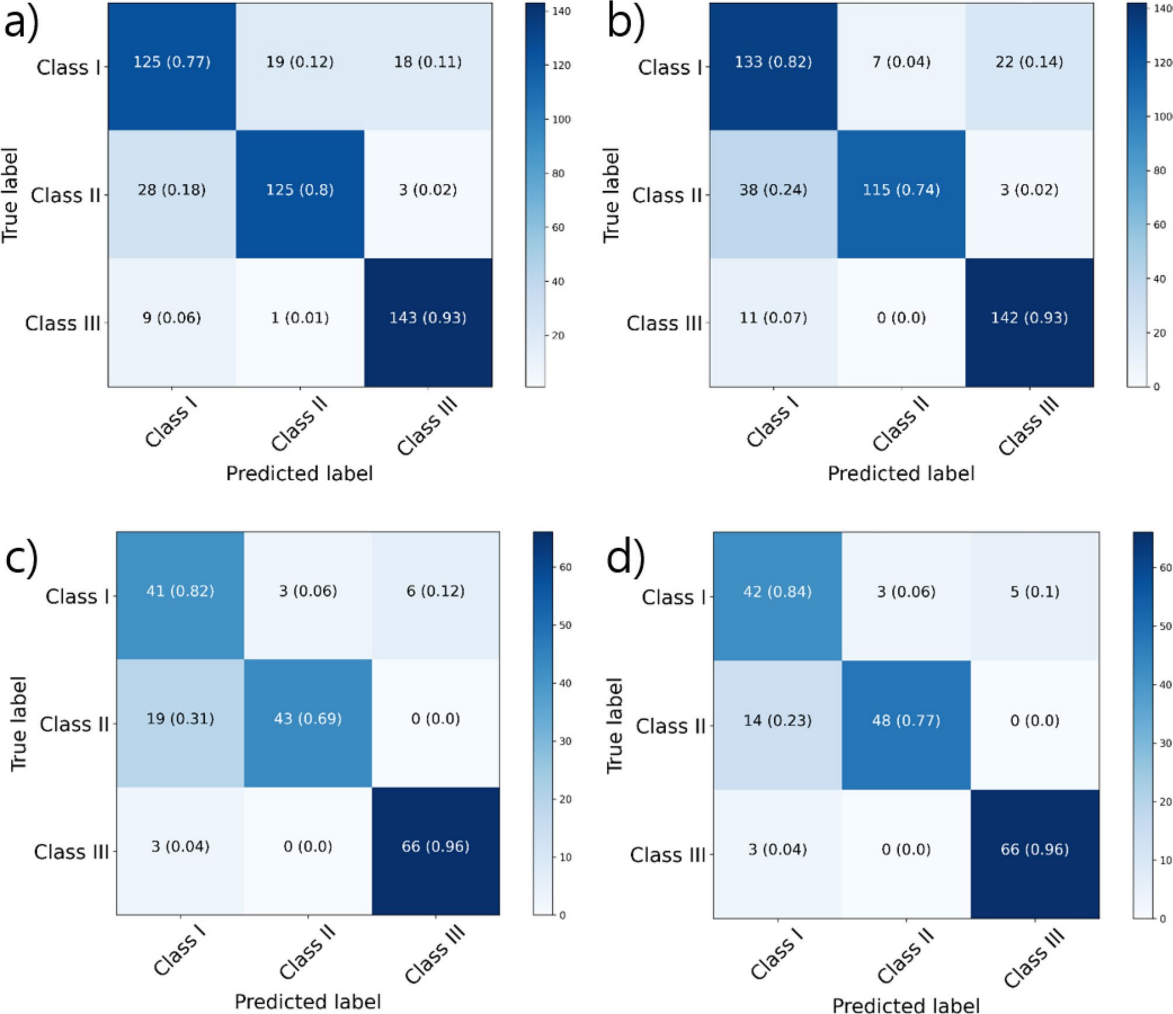
Confusion matrices of classification task for internal (**a,b**) and external (**c,d**) datasets. Left: performance of trained model using only the real dataset. Right: performance of trained model using the real dataset and synthesized images.

The classification performances of the model trained using only the class imbalanced real dataset and that using the dataset balanced by the addition of synthesized images were tabulated in Table 3. In the internal dataset, overall accuracies were 83.4 and 84.9%, respectively. In the external dataset, they were 82.9 and 86.2%, respectively. The accuracy increased by 1.5 and 3.3% for internal and external datasets, respectively. Figure 4 shows the confusion matrices for both the internal and external test sets.

**Table 3:** Accuracies of the classification model for internal and external test sets.

	Real images	+ Generated images
**Internal test set**		
Overall	0.8344	**0.8493**
Each class		
Class I	0.8429	**0.8556**
Class II	0.8917	**0.9108**
Class III	**0.9342**	0.9321
**External test set**		
Overall	0.8287	**0.8619**
Each class		
Class I	0.8287	**0.8619**
Class II	0.8785	**0.9061**
Class III	0.9503	**0.9558**

Training using only the real dataset is indicated by *Real images* and training using the real dataset with generated images is indicated by + *Generated images.*

## Discussion and conclusions

We generated highly realistic cephalometric X-ray images using a PGGAN model. The image Turing test showed that the specificity of Group 1 was significantly lower than its sensitivity. This indicates that the non-orthodontists’ group could not discriminate the synthesized images, whereas for the orthodontists’ group, it was relatively easy to find artifacts in the synthesized images. In addition, the sensitivities of Groups 1 and 2 were not considerably different. This result indicates that non-orthodontists and orthodontists had similar difficulties to discriminate the image as real.

**Figure 5:**
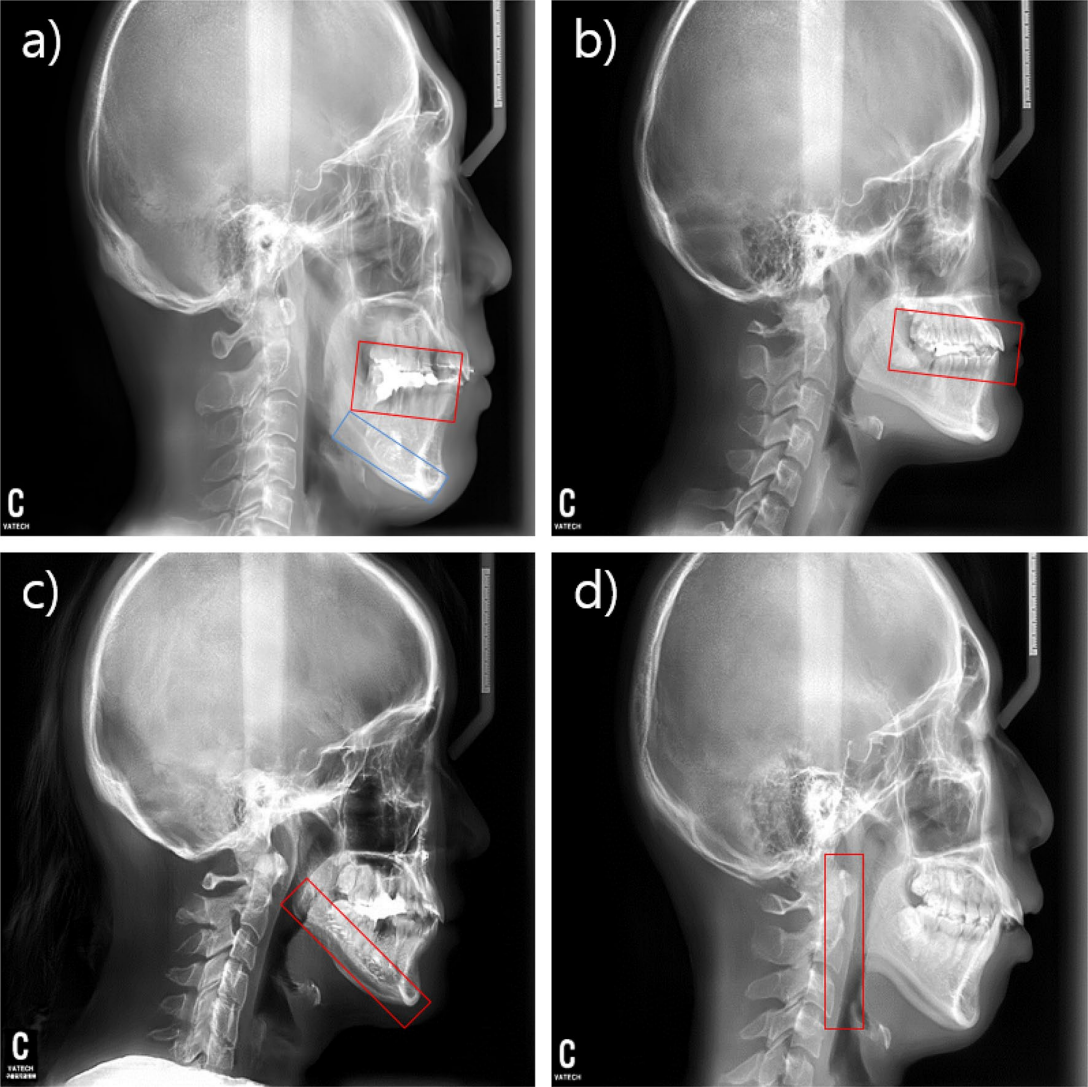
Regions of interest for the most prominent difference features. (**a**) Overlapped teeth region (red color) and radiopaque line (blue color). (**b**) Occlusal-plane point. (**c**) Cortical line of the mandibular. (**d**) Ramal planes.

The most prominent difference between the real and synthesized cephalometric images was in the teeth region. In the synthesized images, the teeth frequently overlapped each other; thus, their anatomical structure could not be clearly distinguished (see red box in Fig. 5a). In addition, the radiopaque line at the cortical bone was artificial in most of the synthesized images (see blue box in Fig. 5a). Group 2 was familiar with cephalometric images, thus they could easily use these features to identify the synthesized images. Group 1 had difficulties distinguishing between the real and synthesized images.

Most of the landmark points identified by the orthodontists had no significant differences between them. Because the landmark positions are identified by the relative positions of anatomical structures, the differences were evaluated by dividing them into the horizontal and vertical axes of the Cartesian plane. Using this metric, 29 landmark points out of 42 showed less than a 2 mm difference in the Cartesian plane. For point A at the maxilla and point B at the mandible regions, the differences in the horizontal direction were smaller than those in the vertical direction. These points were mainly used to evaluate the anterior–posterior relationship. In contrast, the anterior and posterior nasal spine points, which are important for identifying the palatal plane, had smaller differences in the vertical direction than in the horizontal direction. This indicates that the difference is not random but occurs systematically depending on the positional definitions of the landmarks.

The landmark with the largest difference between the orthodontists’ definitions was the occlusal-plane point (see red box in Fig. 5). The difference in the point’s horizontal direction was 5.87 ± 2.43 mm and that in the vertical direction was 0.76 ± 0.54 mm. This point is located at the center of the occlusal plane, which is defined by the position of the first premolar and thus can be identified along the horizontal direction. In the synthesized image, although the structure of the first premolar was unclear and had artifacts, the occlusal plane point was not affected in the vertical direction. Consequently, the occlusal plane point had large horizontal differences between the orthodontists' definitions; however, this artifact did not affect the slope of the occlusal plane.

Furthermore, the synthesized cortical lines (see red box in Fig. 5c) of the mandibular and ramal planes (see red box in Fig. 5d) were straight, not curved. This increased the differences of the corpus left in the horizontal direction and the ramus down in the vertical direction, where the errors at the mandibular plane and ramal plane were relatively decreased. Among the landmarks on soft tissue, the soft-tissue pogonion showed the largest difference between the orthodontists’ definitions. This is because the shape of the chin was flat in the synthesized image and the tissue contrast was too dark to identify the landmark. However, this difference is comparable with the inter-examiner error of Hwang et al.^22^.

Moreover, synthesized images were evaluated for classification task. Synthesized images were added to a real dataset for balancing the number of images in each class. The classification performance was increased for both internal and external test sets compared with the performance of the trained model using only the class imbalanced real dataset. This indicates that synthesized images have clinical information of skeletal pattern. In this study, the smallest number of images was used for balancing the real dataset. The accuracy could be further increased if more synthesized images were added.

The succession of the downstream task indicates important meaning from a following point of view. In the medical field, various occlusal patterns and imbalanced dataset between normal and abnormal datasets caused misclassification for deep learning based artificial intelligence system. Therefore, GAN based augmentation technique as shown in this work to accurately classify the various kinds of normal structure should be needed. Otherwise, anomaly detection technique also has been studied to overcome the extreme imbalanced dataset between normal and abnormal ^23^. This technique trains only normal dataset using GAN under the assumption that the abnormality does not generated. After training, if one inserts an abnormal image to the GAN, it generates normal images excluding abnormalities. The abnormality is then automatically detected by subtracting generated image from inserted abnormal image. Thus, the GAN based anomaly detection in cephalometric images will also be an important field and our work verified the GAN performance in advance.

This study has several limitations. First, because the image Turing test was conducted using limited-resolution images, it should be repeated by synthesizing full high-resolution images (i.e. 1955 × 2360 pixels), which are commonly used in the clinical field. Second, although many cephalometric images were used for this study, the data distribution was not known in terms of anatomic variation, which results in limitations of synthesizing diverse variations. In addition, the aspect ratio was not considered when resizing the cephalometric image to 1024 × 1024 pixels for GAN training. Because relative position and angle between the landmarks are important for orthodontic diagnosis, some portions of clinical information could be reduced. Future studies should consider the aspect ratio or cropping of clinically important regions for GAN generation. Finally, comparisons between GANs such as PGGAN, StyleGAN1, StyleGAN2 should be the further performed, as they can be useful for choosing the best model for clinical application.

Although the PGGAN synthesized images show some artifacts such as in the teeth region, we concluded that the generated images can be used for augmentation of datasets in deep learning and to analyze the positional relations between the set of teeth, basal bone, and skull base through landmark tracing. Although cephalometric images contain complex features such as tooth, tissue, cervical vertebra, and devices, the generated images were highly realistic, as verified through various evaluation methods presented in this study. Those evaluations indicate it was difficult to distinguish between real and synthesized images. Furthermore, classification results of skeletal patterns indicated that the synthesized images contain clinical information to improve the classification accuracy and thus have potential to be applicable to various deep learning studies. In future studies, we expect to improve the artifacts in the cephalometric images by training the GAN with more datasets that contains diverse ranges of anatomic features.

## Supplementary Information

Supplementary Information.

## Data Availability

The datasets are not publicly available because of restrictions in the data-sharing agreements with the data sources. Ethics approval for using the de-identified slides in this study will be allowed upon request to the corresponding authors.
