# Author Correction: Realistic high-resolution lateral cephalometric radiography generated by progressive growing generative adversarial network and quality evaluations

**DOI:** 10.1038/s41598-021-96793-8

**Published:** 2021-08-26

**Authors:** Mingyu Kim, Sungchul Kim, Minjee Kim, Hyun‑Jin Bae, Jae‑Woo Park, Namkug Kim

**Affiliations:** 1 Department of Convergence Medicine, Asan Medical Center, College of Medicine, University of Ulsan, 88 Olympic‑ro 43‑gil, Songpa‑gu, Seoul, 05505 Republic of Korea; 2 Department of Biomedical Engineering, Asan Medical Institute of Convergence Science and Technology, Asan Medical Center, College of Medicine, University of Ulsan, Seoul, Republic of Korea; 3 Department of Orthodontics, Kooalldam Dental Hospital, 1418 Kyoungwondaero, Bupyong‑gu, Incheon, 21404 Republic of Korea; 4 Department of Radiology, Asan Medical Center, College of Medicine, University of Ulsan, Seoul, Republic of Korea

Correction to: *Scientific Reports* https://doi.org/10.1038/s41598-021-91965-y, published online 15 June 2021

The original version of this Article contained an error in the Methods section, under the subheading ‘Training-data collection’, where

“A total of 19,152 cephalometric images of patients who received orthodontic treatment in 2019 were obtained (institutional review board (IRB) No: P01-202011-21-032) from the Kooalldam Dental Hospital in Korea.”

now reads:

“A total of 19,152 cephalometric images of patients who received orthodontic treatment between 2009 and 2019 were obtained (institutional review board (IRB) No: P01-202011-21-032) from the Kooalldam Dental Hospital in Korea.”

The original Article has been corrected.

