# Supplementary material for: Realistic high-resolution lateral cephalometric radiography generated by progressive growing generative adversarial network and quality evaluations
Source: Sci Rep. 2021 Jun 15;11:12563. doi: 10.1038/s41598-021-91965-y (PMC8206205; doi:10.1038/s41598-021-91965-y)
Supplement: Supplementary file 1 — Supplementary Information. [file 41598_2021_91965_MOESM1_ESM.pdf]

Realistic High-resolution Lateral Cephalometric Radiography Generated by Progressive Growing Generative Adversarial Network and Quality evaluations

Mingyu Kim, PhD; Sungchul Kim, Minjee Kim, Hyun-Jin Bae, PhD; Jae-Woo Park, DDS, PhD, and Namkug Kim, PhD

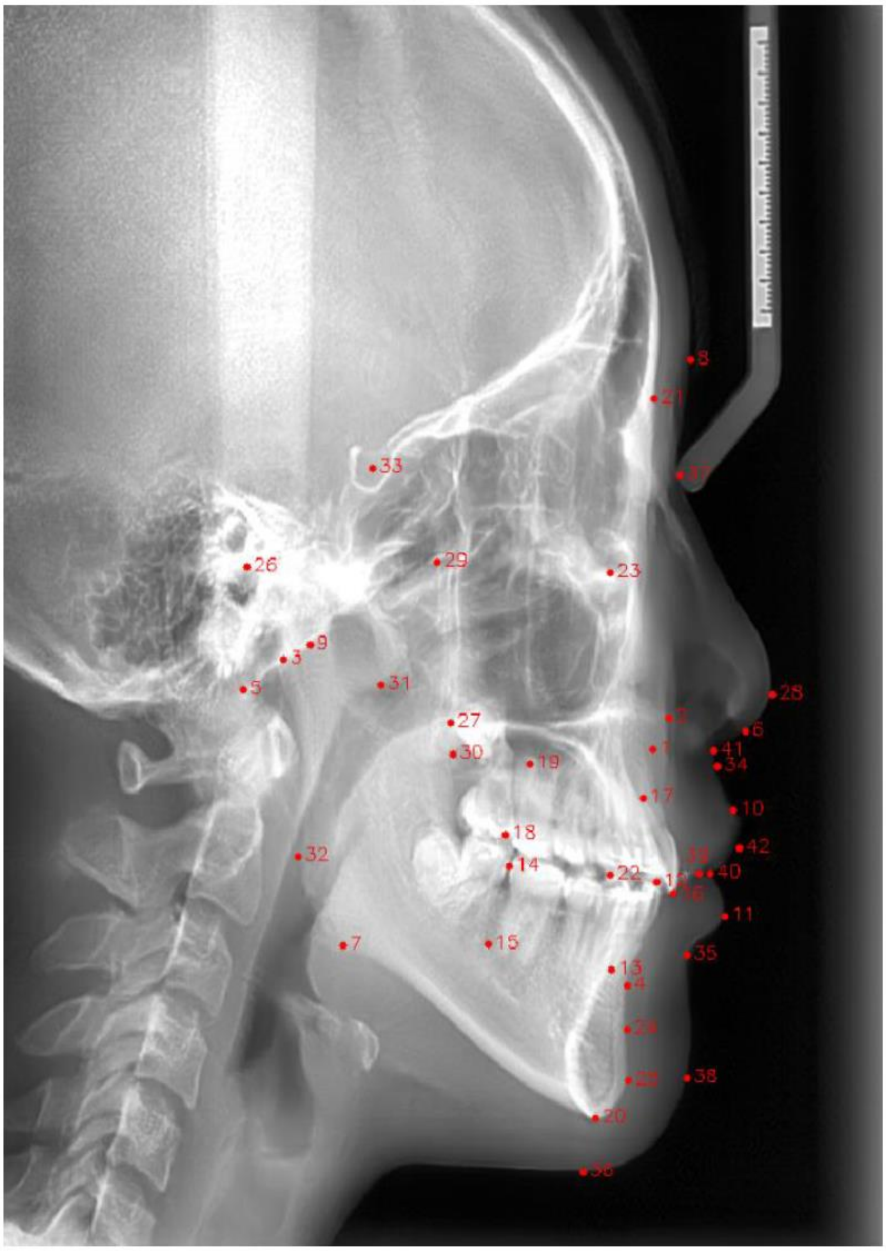

Appendix Figure 1. Forty-two landmarks traced by an orthodontist on a cephalometric image.

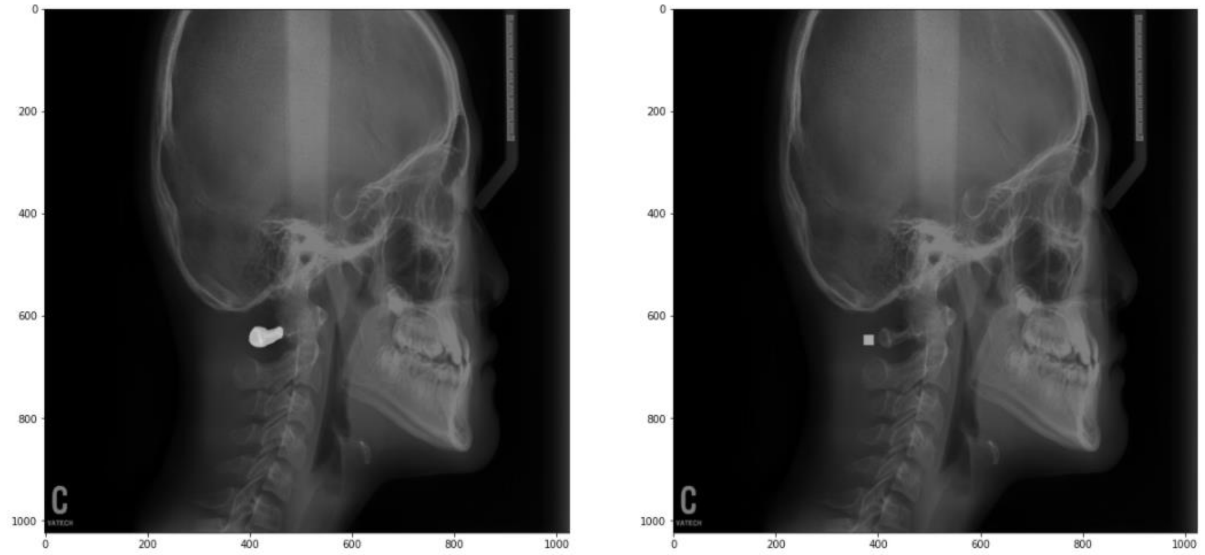

Appendix Figure 2. Example of segmentation for signal-to-noise calculation. Left: segmentation region of signal part at the posterior arch of the first cervical vertebra. Right: segmentation of noise part (10 × 10 pixel) at the posterior direction of the posterior arch.

Appendix Table 1. Landmark names and their corresponding indices, as labeled in Appendix Figure 1.

Index	Name	Index	Name
**1**	A-Point	**22**	Occlusal plane point
**2**	Anterior nasal spine	**23**	Orbitale
**3**	Articulare	**24**	PM
**4**	B-point	**25**	Pogonion
**5**	Basion	**26**	Porion
**6**	Columella	**27**	Posterior nasal spine
**7**	Corpus left	**28**	Pronasale
**8**	Glabella	**29**	Pterygoid
**9**	Hinge axis	**30**	R1
**10**	Labrale superius	**31**	R3
**11**	Lower lip	**32**	Ramus down
**12**	Mandible 1 crown	**33**	Sella
**13**	Mandible 1 root	**34**	Soft tissue A
**14**	Mandible 6 distal	**35**	Soft tissue B
**15**	Mandibl 6 root	**36**	Soft tissue menton
**16**	Maxilla 1 crown	**37**	Soft tissue nasion
**17**	Maxilla 1 root	**38**	Soft tissue pogonion
**18**	Maxilla 6 distal	**39**	Stmi
**19**	Maxilla 6 root	**40**	Stms
**20**	Menton	**41**	Subnasale
**21**	Nasion	**42**	Upper lip

Appendix Table 2. Assessment of the image Turing tests for each reader considering all 100 images.

Group	Readers	Accuracy (%)	Sensitivity (%)	Specificity (%)
**Group 1**	Dental student 1	56.0	74.0	38.0
	Dental student 2	46.0	52.0	40.0
	Non-orthodontic resident 1	44.0	84.0	4.0
	Non-orthodontic resident 2	60.0	56.0	64.0
	Non-orthodontic specialist 1	45.0	72.0	18.0
	Non-orthodontic specialist 2	44.0	66.0	22.0
**Group 2**	Orthodontic resident 1	68.0	72.0	64.0
	Orthodontic resident 2	45.0	70.0	20.0
	Orthodontic specialist 1	56.0	96.0	16.0
	Orthodontic specialist 2	54.0	62.0	46.0
	Orthodontic specialist 3	70.0	56.0	84.0
	Orthodontic specialist 4	92.0	90.0	94.0
	Orthodontic specialist 5	85.0	82.0	88.0
